# Ion Transport Study in CS: POZ Based Polymer Membrane Electrolytes Using Trukhan Model

**DOI:** 10.3390/ijms20215265

**Published:** 2019-10-23

**Authors:** Shujahadeen B. Aziz, Wrya O. Karim, M. A. Brza, Rebar T. Abdulwahid, Salah Raza Saeed, Shakhawan Al-Zangana, M. F. Z. Kadir

**Affiliations:** 1 Prof. Hameeds Advanced Polymeric Materials Research Lab., Department of Physics, College of Science, University of Sulaimani, Sulaimani 46001, Iraq; mohamad.brza@gmail.com (M.A.B.); rebar.abdulwahid@univsul.edu.iq (R.T.A.); 2 Komar Research Center (KRC), Komar University of Science and Technology, Sulaimani 46001, Iraq; 3 Department of Chemistry, College of Science, University of Sulaimani, Sulaimani 46001, Iraq; wrya.karim@univsul.edu.iq; 4 Department of Manufacturing and Materials Engineering, Faculty of Engineering, International Islamic University of Malaysia, Kuala Lumpur, Gombak 53100, Malaysia; 5 Department of Physics, College of Education, University of Sulaimani, Sulaimani 46001, Iraq; 6 Charmo Research Center, Charmo University, Sulaimani 46001, Iraq; salah1966sh@gmail.com; 7 Department of Physics, College of Education, University of Garmian, Kalar 46021, Iraq; shakhawan.al-zangana@garmian.edu.krd; 8 Centre for Foundation Studies in Science, University of Malaya, Kuala Lumpur 50603, Malaysia; mfzkadir@um.edu.my

**Keywords:** polymer blends, impedance study, dielectric properties, electric modulus study, loss tangent peaks, ion transport parameters, Trukhan model

## Abstract

In this work, analysis of ion transport parameters of polymer blend electrolytes incorporated with magnesium trifluoromethanesulfonate (Mg(CF_3_SO_3_)_2_) was carried out by employing the Trukhan model. A solution cast technique was used to obtain the polymer blend electrolytes composed of chitosan (CS) and poly (2-ethyl-2-oxazoline) (POZ). From X-ray diffraction (XRD) patterns, improvement in amorphous phase for the blend samples has been observed in comparison to the pure state of CS. From impedance plot, bulk resistance (R_b_) was found to decrease with increasing temperature. Based on direct current (DC) conductivity (σ_dc_) patterns, considerations on the ion transport models of Arrhenius and Vogel–Tammann–Fulcher (VTF) were given. Analysis of the dielectric properties was carried out at different temperatures and the obtained results were linked to the ion transport mechanism. It is demonstrated in the real part of electrical modulus that chitosan-salt systems are extremely capacitive. The asymmetric peak of the imaginary part (M_i_) of electric modulus indicated that there is non-Debye type of relaxation for ions. From frequency dependence of dielectric loss (ε″) and the imaginary part (M_i_) of electric modulus, suitable coupling among polymer segmental and ionic motions was identified. Two techniques were used to analyze the viscoelastic relaxation dynamic of ions. The Trukhan model was used to determine the diffusion coefficient (*D*) by using the frequency related to peak frequencies and loss tangent maximum heights (tanδ_max_). The Einstein–Nernst equation was applied to determine the carrier number density (*n*) and mobility. The ion transport parameters, such as *D*, *n* and mobility (*μ*), at room temperature, were found to be 4 × 10^−5^ cm^2^/s, 3.4 × 10^15^ cm^−3^, and 1.2 × 10^−4^ cm^2^/Vs, respectively. Finally, it was shown that an increase in temperature can also cause these parameters to increase.

## 1. Introduction

Solid polymer electrolytes (SPEs) are of particular relevance to devices that are technologically significant, such as batteries and fuel cells. To be scaled to an industrial level, one of the important criteria is achieving a large number of free ions that contribute to the net charge transport in liquid state materials [1]. Previously, it has been shown that polymers can be used as insulators and structural materials. Since 1975, more developments have been achieved that use polymers as ion conductors from the combination of suitable salts, i.e., enhancing their ionic conductivities. Manipulation of polymer electrolytes has been constantly carried out in order to improve three main characteristics, which are ionic conductivity, thermal stability, and mechanical strength [2]. In the period ahead, research and development on SPEs will show that they can readily replace the conventional organic sol–gel electrolytes in accordance with dimensional stability, electrochemical durability, processability, flexibility, safety, and long life [3]. Since the1970s, much attention has been given to the study of SPEs. The main motivation in this field is the pioneer works of Wright et al. and Armand et al., which were carried out on ionically conducting polymer-salt mixtures and polymer-salt complexes, respectively [4,5]. Chitosan as a derivative of chitin was used in the present study due to its invaluable properties, such as natural abundance and low-cost biopolymer. Chitosan can act as a host for ionic conduction as a result of its structural composition. It is known that a weakly alkaline polymer electrolyte can be produced as a consequence of dissolving a salt in the polymer matrix. Blending chitosan membranes can significantly improve the thermal/chemical stability while achieving a satisfactory mechanical strength. Moreover, the presence of hydroxyl and amino groups on the backbone structure enable chitosan membranes to have a relatively high level of hydrophilicity, which is essential for operation in polymer electrolyte membrane fuel cells [6,7]. It is widely recognized that a polymer that contains an electron donor group can have the ability to dissolve inorganic salts with low lattice energy through weak coordination bonds to form polymer electrolytes. Nearly all SPE membranes, which contain silver salts, were fabricated for the facilitated olefin transport and poly (2-ethyl-2-oxazoline) (POZ) was used as a polymer solvent to dissolve silver salts [8,9,10]. The monomer of POZ containing O and N atoms is responsible for complexation in POZ based electrolytes [11,12]. POZ can serve as an efficient host polymer in synthesizing polymer electrolytes because of the presence of an electron donor group in the backbone, which is very important for making polymer blends. The blending methodology of two or more polymers has inspired many researchers to improve the chemical and physical properties of individual polymers. It is clearly defined that ionic conduction in polymer electrolytes can be enhanced via increasing the amorphous region [13,14,15,16]. As previously confirmed, it is likely that the mechanical properties and/or structure stability of the material can be manipulated towards improvement through the polymer blending methodology [14,15,16,17]. The obtained blended polymer systems provide more sites for ionic complexation to occur, which facilitates ionic conduction compared to single polymer ones [14,15,16,17,18]. In the past, various ion transport mechanisms have been discussed [1]. Munar et al. [19] have found three parameters, such as ionic mobility, carrier density, and diffusion coefficient in lithium salts incorporated into PEs using a dielectric spectroscopy-based approach. In fact, the ion transport mechanism in polymer physics is not fully understood yet, and thus, the lack of information about ion transport has encouraged researchers to study ion conducting polymer electrolytes extensively [20,21,22,23,24].

In this work, analysis of ion transport parameters of chitosan (CS): POZ polymer blend electrolytes incorporated with magnesium trifluoromethanesulfonate (Mg(CF_3_SO_3_)_2_) was carried out through the use of Trukhan model. This model can allow us to use the related values of peak frequencies and loss tangent maximum heights (tanδ_max_) to calculate the ion transport parameters, such as mobility (*μ*), diffusion coefficient (*D*), and charge carrier number density (*n*). From the diffusion coefficient and direct current (DC) conductivity values at various temperatures, one can calculate mobility (*μ*) and charge carrier number density (*n*) [19]. Therefore, in the present work, the main objective isto estimate ion transport parameters in CS: POZ based SPEs using the Trukhan model.

## 2. Results and Discussion

### 2.1. XRD Study

**Figure 1:**
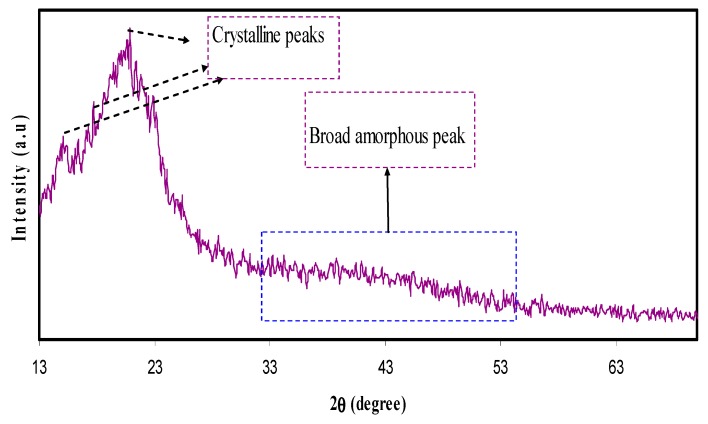
XRD pattern for pure chitosan (CS) film.

**Figure 2:**
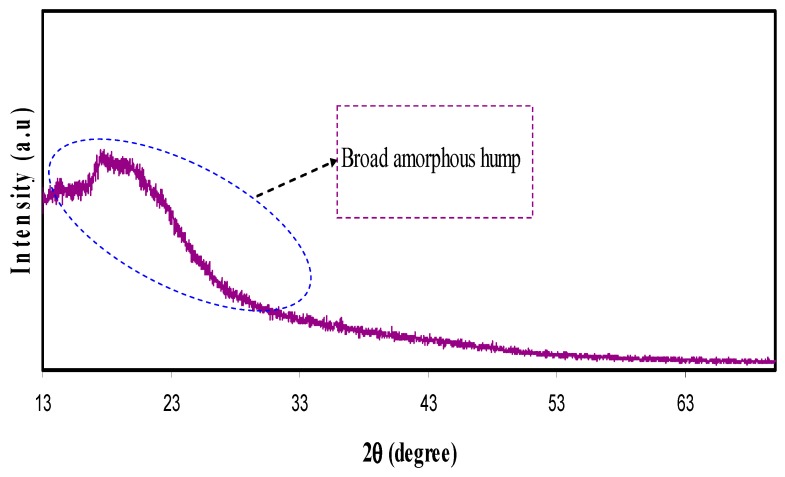
XRD pattern for chitosan: poly (2-ethyl-2-oxazoline) (POZ) polymer blend film.

**Scheme 1:**
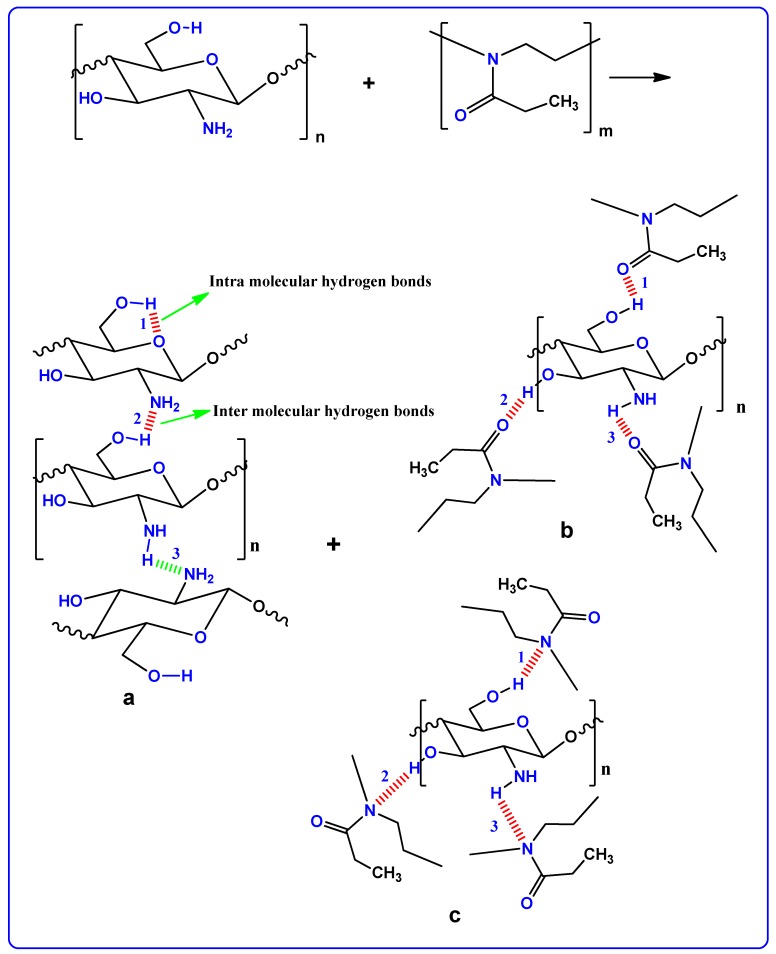
Proposed hydrogen boding between POZ and CS polymers.

Polymer materials are recognized as either being amorphous or semi-crystalline [25]. The crystallinity of polymer structure can be predicted only when a precise method is in place. This not only helps in identifying the molecular structure of a crystalline polymer, but also in comprehending and rationalizing the inherent features of polymeric materials that significantly affect crystal packing [26]. The XRD pattern of pure CS is shown in Figure 1. In the pure state of CS membrane, two peaks can be identified at 14.5° and 20.9°, owing to the crystalline domain of CS polymer. These two peaks are pertinent to two distinct kinds of crystals [27,28]. The first peak that occurs at 14.5° is related to crystals with a unit cell of a = 7.76, b = 10.91, c = 10.30 (Å), and β=90°. Here, the unit cell has two monomer units across the major chain axis. The second peak at 20.9° is related to the crystal within the chitosan membrane. The lattice parameters of the unit cell of crystal related to θ = 20.9°are as follows: a = 4.4, b = 10.0, c = 10.30 (Å), and β = 90° [29]. It can be noticed in Figure 1 that the wide X-ray peak is at approximately 2θ = 41°. It is evident in the previous studies that the wide peaks within the XRD patterns of pure CS polymers are created because of the inter-chain segment scattering as they are in the amorphous state [30]. It can be seen in Figure 2 that when POZ increases (15 wt.%), crystallinity of CS considerably decreases. The reason for this is the creation of hydrogen bonding (H-bonding) between CS and POZ matrices. The creation of hydrogen bonding among CS and POZ monomers disrupts the inter chain hydrogen bonding in the CS host polymer and thus results in less crystallinity. H-bonding is known to occur after the interaction of electron-deficient hydrogen with an area of high electron density. H-bonding can be demonstrated in the form of an intermolecular interaction as X–H…Y, where X and Y indicate electronegative elements and Y contains one more solitary electron pair. This means that X and Y can be signified as F, O, and N atoms [31]. The molecular structure of POZ (refer to Scheme 1) makes it obvious that the POZ monomer contains O and N atoms, which allows it to form H-bonding [11,12]. The hydrogen-bonding is known to be a secondary force, which is weak in comparison to the primary bond within the molecules, such as covalent bonds and other polar bonds. However, this H-bonding is stronger compared to the vander Waals interaction [31]. It is evident from Scheme 1a that around three hydrogen bonds can be created among CS monomers (two -OH and one NH_2_). This means that a chitosan monomer can form three hydrogen bonds at the same time. On the other hand, a POZ monomer has two sites for hydrogen bonding. Therefore, chitosan has strong inter-molecular hydrogen bonding compared to POZ. It is clear from Scheme 1b,c that three hydrogen bonds can be developed between CS and POZ monomers, and thus, the inter-molecular hydrogen bonds between CS monomers can be disrupted. Consequently, a decrease in crystallinity is expected in CS: POZ polymer blends as a result of the loss of intermolecular hydrogen bonds among CS monomers. It can be implied by the broadness of the X-ray peak of the CS: POZ blend that there is greater inter-chain spacing in the blend sample in comparison to that of the CS polymer. The extended inter-chain spacing in the blend makes it very easy to apply dipole reorientation to the field, which leads to a large dielectric constant in comparison to that of the neat polymers [30]. Greater information can be offered by the dielectric analysis regarding how the electrical properties are influenced by polymer blending.

### 2.2. Impedance and DC Conductivity Study

It is vital to fully explain the charge transfer and ionic transport processes in composite materials from fundamental and technological viewpoints. The impedance spectroscopy is a versatile and a powerful tool in analyzing material properties and making establishment of structure–property correlations [32,33].

**Figure 3:**
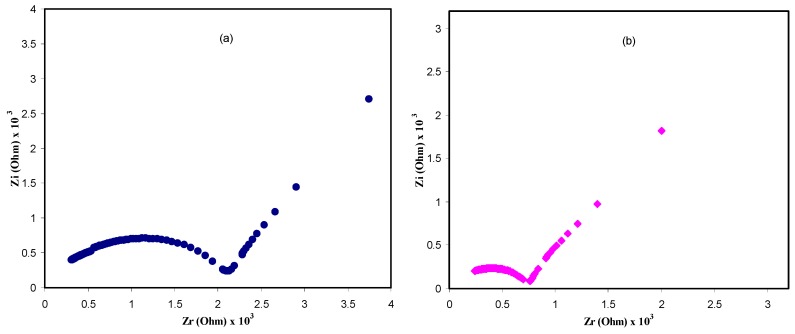

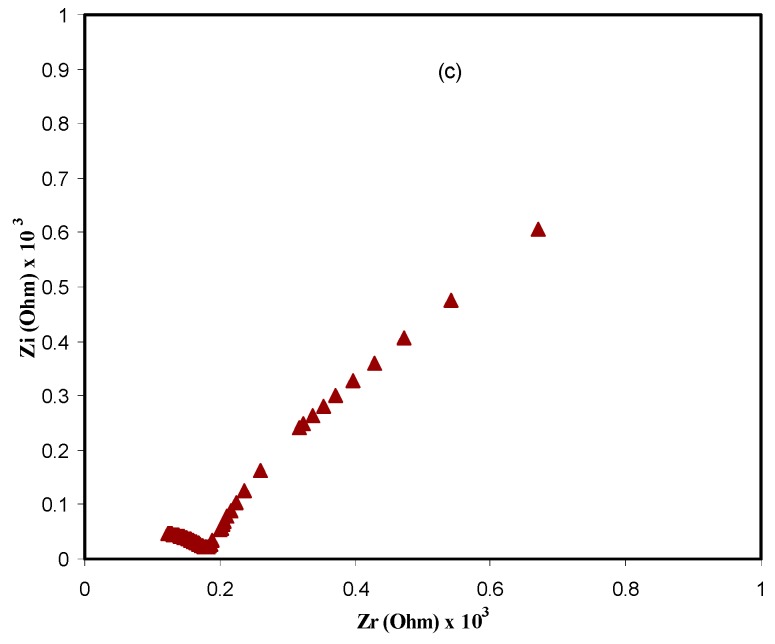
Electrical impedance plots for CS: POZ: MgTf solid polymer blend electrolyte film at (**a**) 303 K, (**b**) 323 K, and (**c**) 343 K.

In Figure 3, the impedance plots (Z_i_ versus Z_r_) for the CS: POZ: Mg(CF_3_SO_3_)_2_ system is shown at different temperatures. It is clearly seen that there is a semicircle in the high frequency region and a linear line (i.e., spike) in the low frequency region. The physical meaning of the semicircle is that this bulk effect reflects a parallel combination of bulk resistance (R_b_) and bulk capacitance of the polymer electrolytes. For the spike explanation, there is an effect of electrode blocking [34,35]. From the data analysis, R_b_ values were extracted from the intercept of high frequency semicircles with the real axis of the impedance plots. Moreover, there is the fact that the complex impedance is dominated by the ionic conductivity when the phase angle approaches zero [36]. Furthermore, the direct current (DC) conductivity (σ_dc_) values were calculated from the following relation:

(1) $$\sigma_{dc}=\left(\frac{1}{R_{b}}\right)\times \left(\frac{t}{A}\right)$$

where *t* and *A* are the thickness and area under study of the sample. In fact, three different cation transport modes have been reported theoretically; firstly, cation diffusion along the polymer chain, secondly, cooperative motion of the cation within the polymer chain, and thirdly, transferring of cations between different polymer chains. It is obvious that the attached ions cause the local polymer motion to slow down and accordingly, the binding of several functional groups to the ion reduces the number of torsional degrees of freedom within the polymer backbone. This all gives rise to the whole polymer-ion complex moving cooperatively [1].

**Figure 4:**
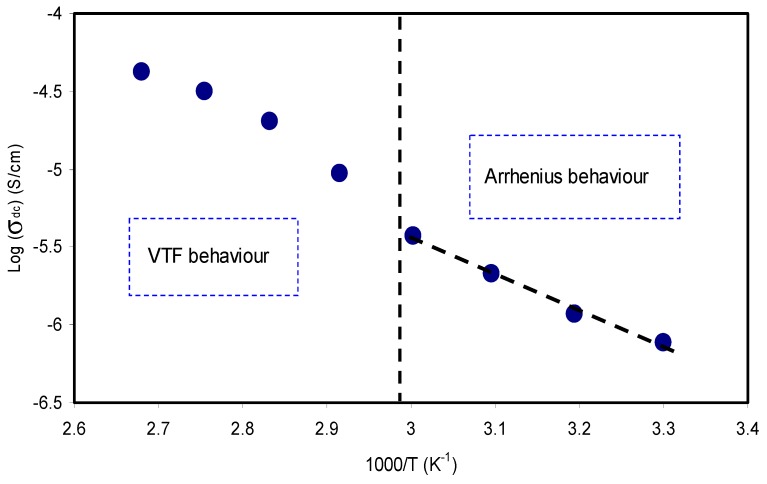
Direct current (DC) ionic conductivity versus 1000/T for CS: POZ: MgTf solid polymer blend electrolyte film.

Obviously, ionic conductivity is one of the key aspects of electrolyte materials to be addressed extensively. It is self-evident that the number of charge carriers and their mobility (i.e., size and electronegativity of an ion) are the two factors that govern conductivity. This is true for polymer electrolyte systems and the mathematical relationship can be described in the following equation [24]:

(2) $$\sigma =\underset{i}{\sum }n_{i}q_{i}\mu_{i}$$

where *n_i_* is the charge carrier′s concentration (i.e., number of charge), *q* is the electron charge, and *µ_i_* is the ions mobility (where *i* refers to the identity of the ion) [20]. It is clear from Equation (2) that the ionic conductivity (σ) increases as the charge carrier concentration (*n*) and/or the ionic species mobility increase in the system. Indeed, the ionic mobility is determined by the three main factors, electronegativity, size of ions, and interaction between polymers and ions. In comparison, the mobility of smaller ions (Li^+^ and/or Mg^2+^) is higher than that of larger ions (Na^+^ and/or Zn^2+^) in the polymer electrolyte [37]. The careful choice of SPEs in a certain application of an electrochemical device depends on the σ_dc_ value. Figure 4 shows the direct current(DC) conductivity as a function of 1000/T for the CS: POZ: MgTf solid polymer blend electrolyte film. Herein, the relationship between DC conductivity and temperature is explained in the form of the Arrhenius equation for linear parts (T≤ 60 °C) as given below:

(3) $$\sigma_{dc}\left(T\right)=\sigma_{0}\exp\left(-\frac{E_{a}}{K_{B}T}\right)$$

where *σ_o_* is the pre-exponential factor, *E_a_* is the activation energy, and *k_B_* is the Boltzmann constant [38]. This equation shows that the motion of cations is not a result of the molecular motion of the polymer host. Thereby, when the temperature and ionic conductivity data strongly obey the Arrhenius relationship, the mechanism of cation transport can be correlated to the occurrence in ionic crystals, where an ion jumps to the nearest vacant site, causing the direct current (DC) ionic conductivity to reach its highest value [39]. Studies have confirmed that ion transport mainly occurs in the amorphous regions. However, while the conduction mechanisms are not fully understood yet, it is widely recognized that cations are interrelated to functional groups of the host polymer chains. It can move through re-coordination along the polymer backbone [40]. The activation energy was calculated and was found to be 0.612 eV for the linear region. The activation energy can be considered as an energy barrier that the ion needs to overcome for a successful jump between the sites [27]. It can be seen that at higher temperatures (T > 333 K) the pattern of DC conductivity vs 1000/T is almost curvature rather than linear behavior. In this case, the Vogel–Tammann–Fulcher (VTF) model is applicable to interpret the behavior of DC conductivity [41]. This is means that ion transport occurs with the help of polymer chain motion. To clarify, the glass transition temperature (T_g_) of POZ is as low as 63 °C [11], whereas T_g_ for CS is relatively quite high and found to be above 200 °C [7,42]. Thus, the curvature of DC conductivity is related to the low T_g_ value of POZ. Consequently, blending CS with POZ can be interesting in developing new polymers with relatively high chain flexibility.

In view of the recent advances in polymer electrolytes, the chain in the polymer is folded in the form of cylindrical tunnels, where the cations are situated and coordinated with the functional groups [43,44]. These cylindrical tunnels offer a pathway for the cation movement. Therefore, there is two options for the segmental motion, either the ions are allowed to hop from one site to another site or they are given a pathway to move [45,46]. It is also understood that the ionic motion can take place through the transitional motion/hopping and dynamic segmental motion within the host polymer [46,47,48]. In this case, the pattern of DC conductivity vs 1000/T is almost linear as observed in the present work at a low temperature (T ≤ 333K). As the amorphous phase progressively expands at high temperature, the polymer chain gains faster internal motion, where bond rotation creates segmental motion. This favors the inter-chain and intra-chain ion hopping and thus ions move successively. Accordingly, this makes the conductivity of the polymer electrolyte to be relatively high [47,49,50]. It is also emphasized that the charge carrier density, *n*, depends mainly on both the dissociation energy (*U*) and dielectric permittivity (ε′) of the polymer electrolyte as shown below [51,52,53]:

(4) $$n=n_{0}\exp\left(-\frac{U}{\varepsilon^{\prime }K_{B}T}\right)$$

where *k_B_* and *T* are the Boltzmann constant and temperature, respectively. This equation indicates that there is a correlation between dielectric permittivity (ε′) and charge carriers. It is well-defined that the dielectric constant is equal to the ratio of the material capacitance (C) to the capacitance of the empty cell (C_o_) (ε′ = C/C_o_). Indeed, an increase of dielectric constant causes an increaseof charge concentrations in the electrolyte. As mentioned in Equation (2), conductivity (σ) depends upon both the number of charge carriers (*n*) and the mobility of the ionic species in the system [54]. Furthermore, from Equation (4), the number of charge carriers (*n*) can be increased by increasing the dielectric constant, in other words, an increase of charge carriers leads to increasing dielectric constant. Overall, the conductivity increases with increasing of dielectric constant based on Equation (4). Here, one can conclude that the dielectric constant is an informative parameter in studying the conductivity behavior of polymer electrolytes [20]. From our discussion and interpretation regarding the ion transport and linkage between DC conductivity and dielectric constant ε′, we can realize that ion transport is a complicated process in polymer electrolyte systems [55]. The lack of information about the cation transport mechanism in polymer electrolytes is thought to be one of the main obstacles in achieving a high conducting polymer electrolyte at room temperature [56,57]. Finally, it is clarified that the ion carrier concentration and segmental mobility are not the only factors that determine the conductivity behavior of polymer electrolytes, but also the dielectric constant and ion dissociation energy are of significant importance to ion transport. Study of the dielectric constant at various temperatures as can be seen in later sections and may support the above explanation.

### 2.3. Dielectric Properties and Electric Modulus Analysis

**Figure 5:**
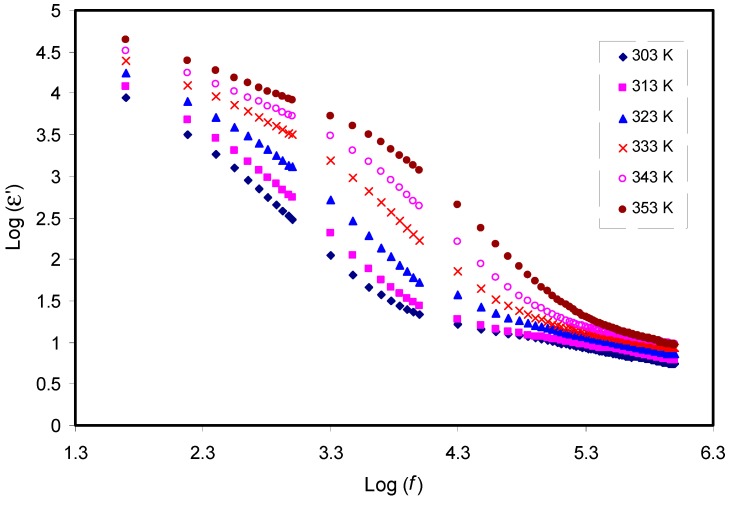
Frequency dependence of dielectric constant (ε′) at selected temperatures for the CS: POZ: MgTf system. Noticeably, the dielectric constant rises with temperature rise.

**Figure 6:**
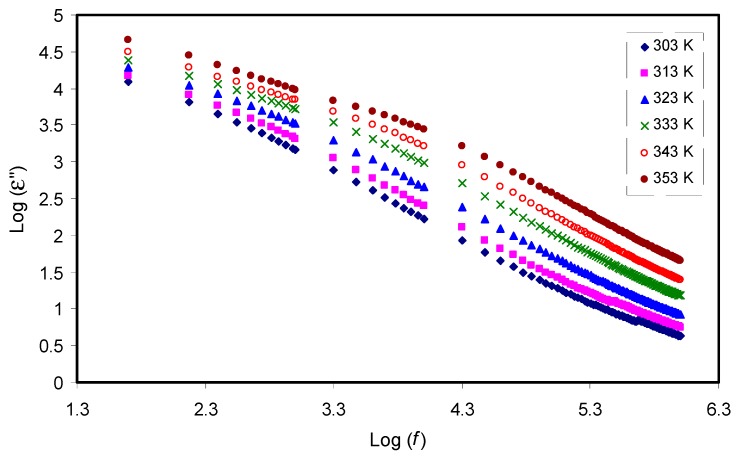
Frequency dependence of dielectric loss (ε′′) at selected temperatures for the CS: POZ: MgTf system. The dispersion in dielectric loss spectra rises with rising temperature.

Figure 5 and Figure 6 show the dielectric constant (ɛ′) and dielectric loss (ɛ′′) as a function of frequency, respectively. As the frequency increases, both ɛ′ and ɛ′′ decrease gradually to a minimum value, becoming almost constant at high frequencies. The obtained high values of both parameters in the low frequency region can be attributed to electrode polarization effect [20,58,59]. This effect is found to result from charge accumulation at the electrode/electrolyte interfacial region [60]. The constant values of both the dielectric constant and dielectric loss at high frequencies can result from the periodic reversal of the high electric field. Consequently, there is no charge accumulation at the interface. The absence of a more interesting observation for the dielectric relaxation peaks within the dielectric loss plot can be due to the mask of polymer relaxation segments by the DC ionic conductivity of charge carriers [22,23,24]. Studying temperature clearly showed that with increasing temperatures, the dielectric constant also increases. This can be clearly observed in Figure 5, which also supports our interpretations given in Section 3.1 for causes of increasing DC conductivity. Once the dielectric constant is obtained, it will be easy for researchers to identify the two following important phenomena: reduction of silver ions to metallic silver particles and electrical percolation in ion conducting solid polymer composites [21,58,61,62,63]. One can note from Figure 5 and Figure 6 that both ε′ and ε′′ values recorded high values at high temperature. This can be explained in terms of the forces that bind the polymer chains together. Obviously, there is an existence of forces within polymer material matrices, which are generally classified into primary (intra-chain) and secondary (inter-chain) forces, that result in the stabilization of polymer structure [23,64]. On the one hand, the primary ones arise from the covalent bond formation (2.2–8.6 eV), which binds the chains of backbone atoms together. On the other hand, there are four types of secondary forces in polymers, which are dipole–dipole bonding (0.43–0.87 eV), hydrogen bonding (0.13–0.30 eV), induced interaction (0.07–0.13 eV), and dispersion interaction (0.002–0.09 eV). These forces possess relatively low dissociation energies; thereby, these forces are extremely susceptible to temperature change compared to their primary counterpart.

**Figure 7:**
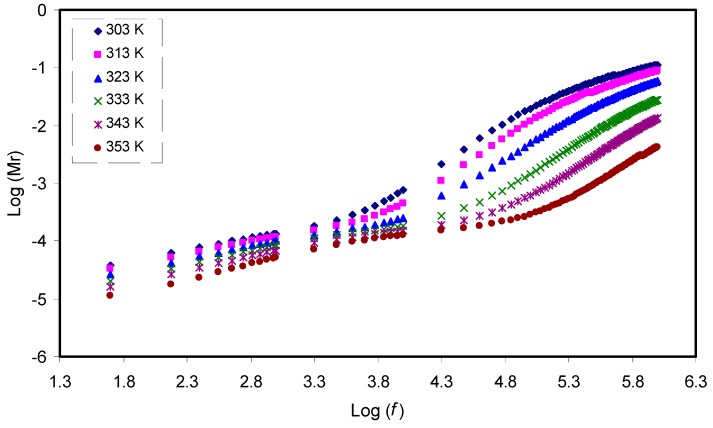
Frequency dependence of the real part (M_r_) of M* for the CS: POZ: MgTfsystem at different temperatures.

**Figure 8:**
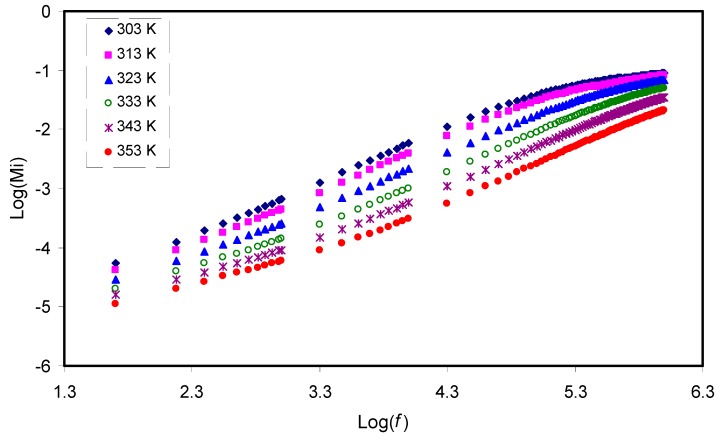
Frequency dependence of the imaginary part (M_i_) of M* for the CS: POZ: MgTf systemat different temperatures.

The electric modulus is the inverse of electric permittivity and can be used to study the dielectric behavior of a polymer resulting from ion relaxation. In this work, suppression of charge accumulation near the electrode is related to the electrode polarization effects [52,65]. Figure 7 and Figure 8 exhibit the real (M_r_) and imaginary (M_i_) components of electric modulus versus frequency, respectively. In contrast to the ɛʹ spectra in Figure 5, detectable peaks, especially for curves at 303K and 313K resulting from conductivity relaxation, are seen in the M_i_ spectra as presented in Figure 8. It is observable that both M_r_ and M_i_ decrease towards low frequencies, indicating the negligibility of electrode polarization influence. In both plots, the curves show long tail features at low frequency at various temperatures that can be due to the suppression of low frequency electrodes/sample double layer effects, arising from the large capacitance establishment [66,67]. In contrast to the ɛ′ spectra in Figure 5, detectable peaks, which result from conductivity relaxation, can be seen in the M_i_ spectra as shown in Figure 8. Previously, it had been confirmed that ɛ′ parameter is always influenced by an ohmic conduction, i.e., DC conductivity, which leads to the hidden loss peaks in the ɛ′ spectra [68]. A peak in the M_i_ spectra indicates the regions where the carrier can move a long distance (left of the peak) or where the carrier is confined (right of the peak) [69]. Viscoelastic relaxation or conductivity relaxation using Argand plots is the well-known method to explain the relaxation dynamic. Studying Argand plots at various temperatures is informative to address the nature of relaxation processes within the present polymer electrolytes.

**Figure 9:**
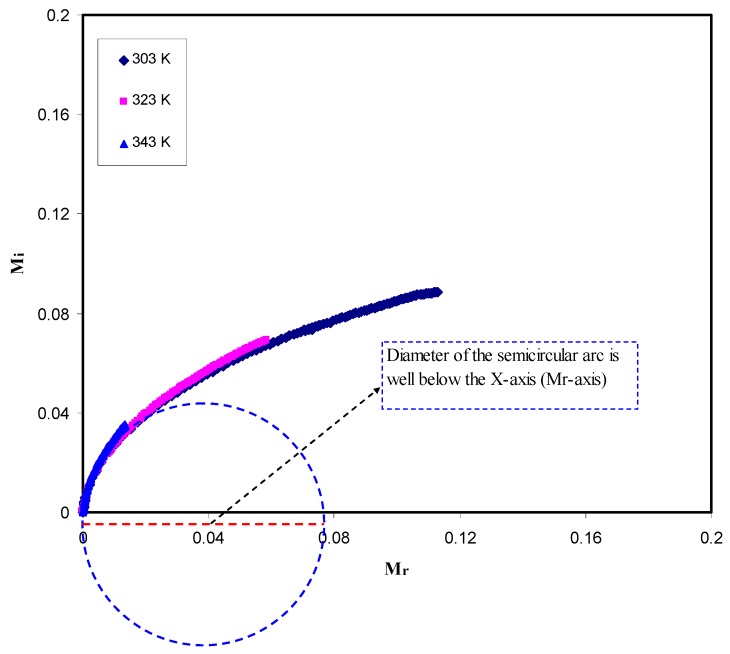
Argand plots for the CS: POZ: MgTf system at different temperatures.

Figure 9 reveals the Argand curves at room temperature. As can be seen from the figure, the diameter of the circles does not coincide with the real axis, which presents (M_r_), and thus the ion transport occurs via the viscoelastic relaxation. The characteristics of the Argand plot are am incomplete half semicircle and arc-like appearance, which cannot be explained by the Debye model, i.e., single relaxation time [70]. Therefore, a distribution of relaxation time is necessary to interpret the experimental data, since more dipoles and ions are activated and contribute to the dielectric relaxation with increasing temperature [7,65,71]. Obviously, with increasing temperature, the Argand distributed data points shift towards the origin. This can be attributed to the increase of conductivity due to the increase of ionic mobility with temperature elevation, and accordingly caused both Z′ and Z′′ to decrease. Mohomed et al. [72] have explained that Argand plots (M_i_ vs. M_r_) may have a complete semicircular shape and in this case, the relaxation could be due to the conductivity relaxation dynamic, i.e., pure ionic relaxation, that is, the polymer segmental motion and ion transport are completely separated or decoupled. On the other hand, if Argand plots (M_i_ vs. M_r_) have an incomplete semicircular shape, the relaxation could be then attributed to viscoelastic relaxation. This implies that ion motion and polymer segmental motion are well coupled. From our Argand plots, the semicircular behavior with diameter well below the real axis can signify that the relaxation process has arisen from the viscoelastic relaxation dynamic. This is means that ion transport is coupled with the polymer segmental motion and thus no peaks can be seen in the dielectric loss spectra as shown in Figure 6. The obtained results can be interpreted on the basis of the fact that the ion transports in SPEs are enhanced by large scale segmental movements of the polymer chain.

### 2.4. Tanδ Spectra and Ion Transport Study

**Figure 10:**
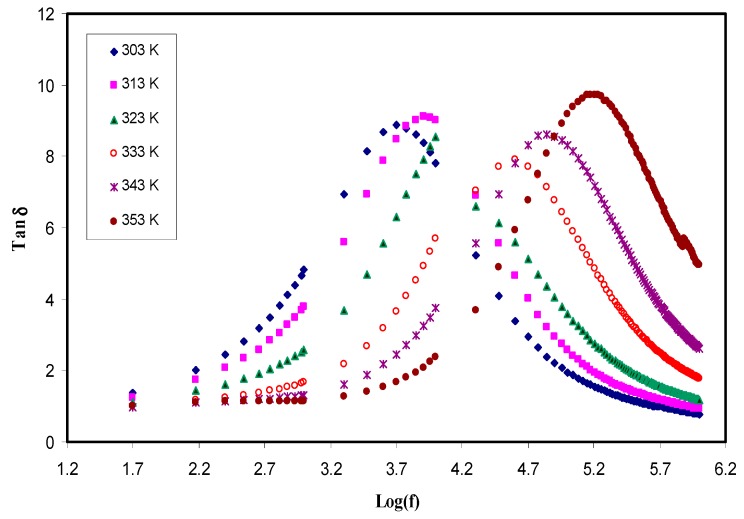
Tanδ plots for the CS: POZ: MgTfsystem at different temperatures.

In polymer electrolyte systems with high electrical conductivity, the peaks at the low frequency relaxation can be masked due to permanent or induced dipoles by polarization relaxation of mobile charged species in the material [73]. To understand the relaxation process, the dielectric loss tangent (tanδ) as a function of frequency has been plotted as shown in Figure 10. The plot shape of tanδ can be interpreted in terms of Koop′s phenomenological principle [74]. Thereby, tanδ increases with increasing frequency, reaching a maximum value, and thereafter decreases with further increasing frequency. On the one hand, at lower frequencies, where tanδ increases, theohmic component of the current increases more sharply than its capacitive component (X_c_ = 1/2π*f*C). On the other hand, at higher frequencies, where tanδ decreases, the ohmic component of current is obviously frequency independent and the capacitive component increases due to the high value of *f* and as a consequence, the value of *X_c_* is small [74,75]. It is worth-noticing that the broad nature of the tanδ peaks suggests that the relaxation process is of non-Debye type [76]. The complex impedance relationship is Z* = *R*-j*X_c_*, where *R* is the real or resistor element and X_c_ is the capacitive element [62]. This function indicates that, at low frequency, the capacitive component becomes very high and as a result, most of the current passes through the resistor element. The tanδ relation (tanδ = ɛ′′/ɛ′) shows the proportionality of tanδ to ɛ′′. Also, from these relationships, one can obtain ɛ′′, which is exactly in proportion to the real part (Z′ or R) of the impedance function, through the following equation:

(5) $$\varepsilon^{\prime \prime }=\frac{Z^{\prime }}{\omega C_{0}\left({Z^{\prime }}^{2}+{Z^{\prime \prime }}^{2}\right)}$$

As the temperature is increased, the loss tanδ peaks shift to the higher frequency side. Figure 10 shows that the position and height of the peaks are increased with increasing the temperature. This is explained on the basis of the fact that the higher temperature makes the charge carrier movement easier and thus capable of relaxation at higher frequencies [77,78]. From all these discussions stated above, it can be realized that both the shape and intensity of tanδ peaks at various temperatures are completely related to the ion transport parameters, such as mobility and diffusion coefficient. From tanδ_max_ and peak frequency, one can extract mobility (*μ*), carrier density (*n*), and diffusivity (*D*) as a function of temperature, which will be discussed in the next sections.

In the analysis of the loss tangent, the shape of the tanδ plots is correlated to both the capacitive and resistive component of the solid electrolytes. Furthermore, it is explained that the shifting of tanδ peaks towards the high frequency side is associated with the thermally activated ions. These findings support our previous explanation for the DC conductivity pattern versus 1000/T. To calculate the charge density, mobility, and diffusion coefficient of the charge carriers, the Trukhan model was employed by analyzing the loss tangent data points. In this model, the diffusion coefficient of cations and anions are assumed to be equal and thus, a simple expression has been used to calculate the diffusion coefficient from the peak appearance in the loss tangent plots. The expression is shown as follows [19]:

(6) $$D=\frac{2\pi f_{max}L^{2}}{32\;tan^{3}\delta_{max}}$$

where L is the sample thickness and all other terms have normal meanings.

**Figure 11:**
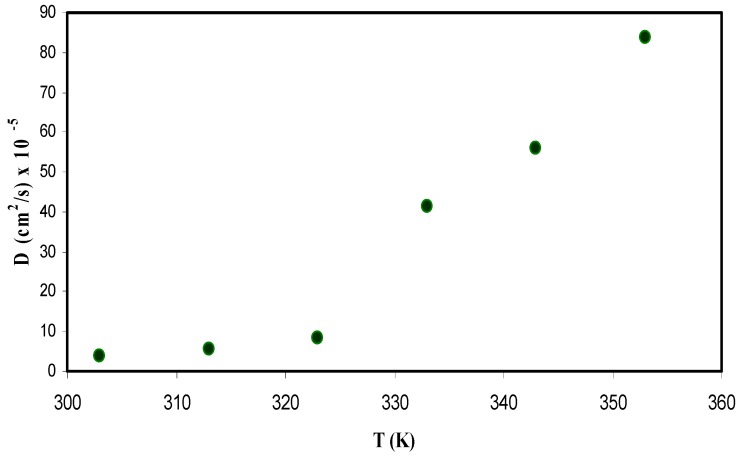
Diffusion coefficient of ions versus temperature for the CS: POZ: MgTfsystem.

Figure 11 shows a plot between diffusion coefficient and temperature. From the plot it is observed that the diffusion coefficient increases non-linearly with the temperature.

Another important parameter, which is in relation to conductivity, is the density of mobile ions. The density number of mobile ions (*n*) can be obtained from the well-known Einstein relation using the following equation [19]:

(7) $$n=\frac{\sigma_{DC}K_{B}T}{De^{2}}=\left(\frac{\sigma_{DC}K_{B}T}{e^{2}}\right)\left[\frac{32\;tan^{3}\delta_{max}}{2\pi f_{max}L^{2}}\right]$$

where σ_dc_ is the DC conductivity and obtainable from impedance plots, *K_B_*, *T*, *e*, and *D* have the usual meaning. From Equation (7), it is obvious that charge carrier density is proportional to cubic of tanδ_max_ value and inversely proportional to the shifting of peak frequencies. It is also clarified that the tanδ_max_ values do not change significantly as there is no considerable change in intensity values; as a consequence, the carrier density becomes almost constant.

**Figure 12:**
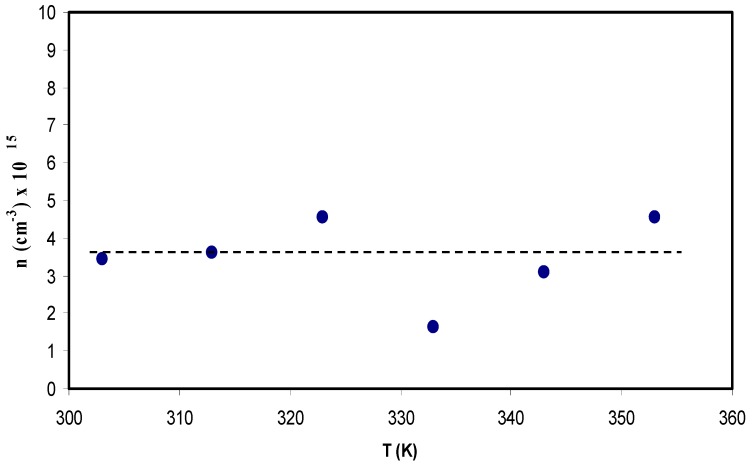
Charge carrier density (*n*) of ions versus temperature for the CS: POZ: MgTf system.

Figure 12 shows charge density (*n*) as a function of temperature. It is seen that the *n* does not change as temperature increases. The Trukhan model allows an estimation of the diffusion coefficient (*D*), mobile ion concentration (*n*), and mobility (*μ*) from the value of (tanδ_max_), where δ is a phase angle. The model is simple in analysis of the data points and there is no need for microscopic information about the distance between adjacent functional groups. However, any increase and decrease of tanδ_max_ intensity directly affects the behavior of carrier density. Chandra et al. [79] have recorded that the value of *n* is found to be in the range of 10^16^ to 10^18^ cm^−3^ for PEO: PVP: AgNO_3_ based SPE systems. Agrawal et al. [80] have studied the mobile ion concentration in the range of 10^15^ to 10^16^ cm^−3^ for hot press PEO: AgNO_3_: SiO_2_ nano-composite system. It is also documented that the conductivity is dependent on both the number of mobile ions and mobility [80]. In the present work, values of the carrier density of (1.5–4.7) × 10^15^ cm^−3^ were recorded and are in good agreement compared to those reported for polymer based solid electrolytes in the literature [79,80].

Ion mobility is also another parameter that can be calculated from the following equation:

(8) $$\mu =\frac{\sigma_{DC}}{en}=\frac{De}{K_{B}T}=\left(\frac{e}{K_{B}T}\right)\left[\frac{2\pi f_{max}L^{2}}{32\;tan^{3}\delta_{max}}\right]$$

where *μ* is the ionic mobility and all other terms have normal meanings.

**Figure 13:**
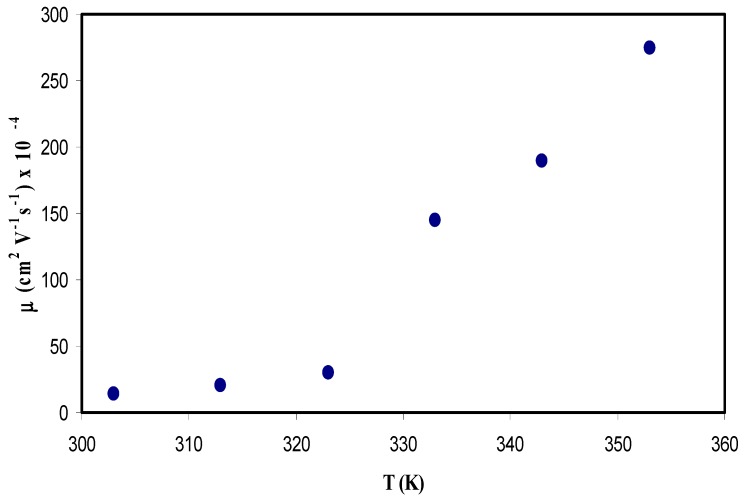
Ion mobility (*μ*) versus temperature for the CS: POZ: MgTf system.

Figure 13 shows the relation between temperature and ion mobility. The dependence of μ on temperature is similar to that in diffusivity. An increase in temperature results in an increase in the mobility, however, this increase does not linearly change. The concept of increasing the ion mobility is supported by the free volume model, in which the temperature increase leads to enlarged free volume in the amorphous phase, which helps ion mobility [75]. For example, the ion mobility values have been found to be 1.5 × 10^−3^ cm^2^/Vs at room temperature, while it is about 2.8 × 10^−2^ cm^2^/Vs at 353 K. Winie et al. [81] have also confirmed such a direct proportionality relationship between ion mobility and temperature. Several research groups have reported results on ion mobility. Majid and Arof [82] recorded the value of mobility (*μ*) in the range 10^−8^ to 10^−6^ cm^2^ V^−1^ s^−1^. Agrawal et al. [80] reported a different value of 10^−3^ cm^2^ V^−1^ s^−1^ for ionic mobility. Moreover, Arya and Sharma [83] reported ion mobility (*μ*) in the range 10^−10^ to 10^−12^ cm^2^ V^−1^ s^−1^. More recently, Patla et al. [84] have also documented ion mobility (*μ*) in the range 1.8 × 10^−4^ to 9.5 × 10^−11^ cm^2^ V^−1^ s^−1^ for polyvinylidene fluoride (PVDF) based polymer nano-composites incorporated with ammonium iodide (NH_4_I) salt. From the results of the present work, it is clear that ion transport parameters can be easily estimated from the Trukhan model. It has been reported that polymers with optical absorption behaviour and high-performance optical properties are important in the application of solar cell devices [85,86,87]. Similarly, fabrication of polymer electrolytes with high ionic conductivity and improved ion transport properties are crucial in the application of electrochemical devices [14,16].

## 3. Materials and Methods

### 3.1. Materials and Sample Preparation

The SPEs based on the CS: POZ blend was fabricated using a conventional solution cast technique. The procedure involves dissolution of 1 g of CS in 80 mL of 1% acetic acid. To prepare the polymer blend, 15 wt.% (0.1764 g) of poly (2-ethyl-2-oxazoline) (POZ) is dissolved in 20 mL of distilled water and added to the CS solution. This solution was continuously stirred with the aid of magnetic stirrer for several hours until a homogeneous viscous blend solution was achieved. Afterwards, 30 wt.% of Mg(CF_3_SO_3_)_2_ salt was added to the CS: POZ solution to obtain an alkaline solution of CS: POZ: Mg(CF_3_SO_3_)_2_ polymer blend electrolyte. Subsequently, the mixture was further stirred until a homogeneous solution was achieved. Finally, the solution was put into a Petri dish and then dried by leaving it to form a film at room temperature. The last step in this procedure was putting it into a desiccator to ensure dryness.

### 3.2. Characterization Techniques

X-Ray Diffraction (XRD) patterns were acquired using an Empyrean X-ray diffractometer, (PANalytical, Netherland) with operating current and voltage of 40 mA and 40 kV, respectively. The samples were irradiated with a beam of monochromatic CuKα X-radiation of wavelength λ = 1.5406 Å and the glancing angle of the X-ray diffraction was in the range of 5° ⩽ 2θ ⩽ 80° withastepsizeof0.1°.

The acquisition of impedance spectra of the films was conducted using a HIOKI 3531 Z Hi-tester in the frequency range of 50 Hz to 1000 kHz and at various temperatures ranging from 303 K to 373 K. The prepared SPE films were cut into small discs of 2 cm in diameter and placed between two stainless steel electrodes under spring pressure. The cell was connected to a computer equipped with customized software to record both real (Z′) and imaginary (Z′′) parts of the complex impedance (Z*) spectra. Consequently, from these data, complex permittivity (ε*) and complex electric modulus (M*) can be then estimated.

## 4. Conclusions

A solution cast technique was used to perform the integration of chitosan (CS) with poly (2-ethyl-2-oxazoline) (POZ). It was shown in the XRD spectra that there was an improvement in the amorphous phase within CS: POZ blend films when compared to pure CS. The broad peak in the CS: POZ blend films compared to the crystalline peaks of the CS polymer confirms this improvement. It can be concluded that a potential technique for studying the diffusion coefficient, mobility, and charge carrier number density in CS: POZ: MgTf solid polymer blend electrolyte film can be the Trukhan model. The calculations carried out in this technique employed peaks in loss tangent spectra. Temperature increases are found to cause a reduction in the bulk resistance and a rise in permittivity. There is also a change found in the position and height of loss tangent peaks verses frequency plots with temperature. The increase in temperatures causes the height of the peaks to shift positively. An Arrhenius type dependence on temperature was exhibited from the DC conductivity; hence, when temperature increases, the conductivity also improved. The increase in the amorphous phase is found to be the reason for such increment in conductivity. Emphasis has been laid on the role of association and disassociation of ionic motion and polymer segmental relaxation. Assessments of dielectric properties were carried out at different temperatures and their findings were related to the ion transport mechanism. It is demonstrated in the real part of the electrical modulus that chitosan-salt mechanisms are very capacitive. According to the asymmetric peak of the imaginary part (M_i_) of the electric modulus, there is a non-Debye form of relaxation for chitosan-salt systems. It was shown by the frequency dependence of dielectric loss (ε′′) and the imaginary part (M_i_)of the electric modulus that there was suitable coupling among polymer segmental and ionic motions. Studies were carried out on the viscoelastic relaxation dynamic of the chitosan-based solid electrolyte using two techniques. The estimation of the diffusion parameter (*D*) was carried out through the Trukhan model by obtaining the frequency related to peak frequencies and loss tangent maximum heights (tanδ_max_). The Einstein–Nernst equation was used to determine the carrier number density (*n*) and mobility. It was found that the ion transport parameters at room temperature are 4 × 10^−5^cm^2^/s, 3.4 × 10^15^ cm^−3^, and 1.2 × 10^−4^ cm^2^/Vs for *D*, *n*, and *μ*, respectively. It was shown that as the temperature increased; there was also an increase in the parameters.
